# Sharing Individual-Level Health Research Data: Experiences, Challenges and a Research Agenda

**DOI:** 10.1007/s41649-017-0029-5

**Published:** 2017-11-28

**Authors:** Phaik Yeong Cheah, Nicholas P. J. Day, Michael Parker, Susan Bull

**Affiliations:** 1 Mahidol Oxford Tropical Medicine Research Unit, Faculty of Tropical Medicine, Mahidol University, Bangkok, Thailand; 2 Centre for Tropical Medicine and Global Health, Nuffield Department of Clinical Medicine, University of Oxford, Oxford, UK; 3 The Ethox Centre, Nuffield Department of Population Health, University of Oxford, Oxford, UK

**Keywords:** Data sharing, Data management, Thailand, Low-income settings, Broad consent

## Abstract

Since January 2016, the Mahidol Oxford Tropical Medicine Research Unit (MORU) has trialled a data-sharing policy where requests to access research datasets are processed through a Data Access Committee. In this paper, we share our experiences establishing data management systems and data-sharing infrastructure including a data-sharing policy, data access committee and related procedures. We identified a number of practical and ethical challenges including requests for datasets collected without specific or broad consent to data sharing and requests from pharmaceutical companies for data to support drug registration applications. We also encountered significant resource constraints which required the development of appropriate human resources and infrastructure. We suggest a research agenda to promote responsible and equitable data sharing while safeguarding the rights and interests of research participants and researchers.

## Introduction

We welcome the International Committee of Medical Journal Editors (ICMJE) position statement on the data-sharing requirements it expects from clinical trial authors (Taichman et al. 2017). The ICMJE does not mandate data sharing but requires authors to include data-sharing statements when reporting results of clinical trials. While we agree that there are many potential benefits from sharing data more widely, we are also aware of concerns about potential harms to primary researchers and data subjects raised by many authors (Bull et al. 2015a).

Since January 2016, the Mahidol Oxford Tropical Medicine Research Unit (MORU) has trialled a data-sharing policy in which requests for access to research datasets are processed through a Data Access Committee (DAC) (Cheah and Day 2017). MORU was established in 1979 as a research collaboration focusing on tropical medicine between Mahidol University in Thailand and the Nuffield Department of Medicine, University of Oxford, in the United Kingdom. The main office and laboratories are located within the Faculty of Tropical Medicine in Bangkok, Thailand, but research is conducted in many different locations both in Southeast Asia and more widely in South Asia and Africa. At any one time, MORU coordinates around 60 to 70 active clinical studies on malaria and neglected diseases such as melioidosis and unexplained fevers. They range from small single-centre studies to large multicentre studies recruiting tens of thousands of participants.

In recent years, MORU has coordinated with some of the largest international studies involving many sites in low-income and hard-to-reach settings (Ashley et al. 2014; Dondorp et al. 2010; Landier et al. 2017; Onyamboko et al. 2014). The majority of the studies coordinated by MORU are sponsored by the University of Oxford and funded by charitable foundations including Wellcome (https://wellcome.ac.uk) and the Bill & Melinda Gates Foundation (https://www.gatesfoundation.org/).

To date, the MORU DAC has received 17 applications, of which 14 have been assessed, and three are currently under review. The process of setting up a data-sharing policies and procedures has been challenging. In this paper, we outline our experiences establishing mechanisms for data sharing and the practical and ethical challenges encountered. We also suggest a research agenda to promote responsible and equitable data sharing while safeguarding the rights and interests of research participants and researchers.

## Data-Sharing Policies and Processes

As discussed by Taichman et al. (2017), requisite mechanisms need to be put in place before sharing of individual participant data can become a norm. In our experience, these mechanisms fall into two main categories: data management and data sharing.

### Data Management and Related Procedures

In 2007, the Clinical Trials Support Group was set up at MORU, within which a data management department was established. There are currently six full time professional data managers who perform data management for all clinical trials, as well as the majority of research studies coordinated by MORU. After a comprehensive review of available data management software, e.g. OpenClinica (http://www.openclinica.com), REDCap (https://www.project-redcap.org/) and a trial period, we purchased MACRO EDC (http://www.infermed.com), a commercial US FDA CFR Part 11 compliant data management software package. Data management standard operating procedures were developed to include procedures such as case report form development, database development and testing, data entry, cleaning and database lock.

These basic data management procedures and infrastructure were a prerequisite for any data-sharing procedures. For data sharing, standard templates for clinical research documents including protocols, information sheets and consent forms were updated to incorporate data sharing. For multicentre studies, it was necessary to engage with collaborators and ensure that clinical trial agreements included provisions for data sharing.

### Data-Sharing Policy and Related Requirements

In January 2016, MORU implemented a data-sharing policy and established the DAC. Applicants complete an application form and applications are considered by the DAC on a case-by-case basis. Application review is informed by a checklist which includes consideration of the objective of a project, the analysis plan, planned outputs and any potential ethical issues. Consideration involves consultation with investigators, relevant collaborators and other experts. In some circumstances, specific conditions of access were implemented, including a requirement for collaboration. In many cases, a formal data access agreement was signed between the University of Oxford and the requesting institution. The main provisions of this data access agreement include using the data only for the purpose stated in the agreement, not transferring data to third parties, and the terms and conditions of termination of the agreement. Once the agreement is in place, the dataset is sent via a secure web-based file transfer system.

Our governance policies and processes for sharing research outputs were informed by an international collaborative study into best practices in sharing individual level data in low- and middle-income settings (LMICs) (Bull et al. 2015a, b; Cheah et al. 2015). This was supplemented by a series of internal consultations with MORU scientists and a review of MORU’s main funders’ policies as well as those of leading journals.

In the collaborative data-sharing study, interviews and focus group discussions were conducted with a range of stakeholders, including researchers, community members and research participants, in Kenya, South Africa, Vietnam, India and Thailand. Respondents in the qualitative study mostly agreed that sharing individual-level data was beneficial in principle. However, many also had important concerns about costs, data quality, participant consent and establishing an effective and trusted approach to data governance. It was felt that potential harms to data subjects, primary researchers and collaborators and also public trust might be best mediated through the adoption of a managed approach. Our data-sharing policy took into account these concerns.

## Practical and Ethical Challenges

This section describes practical and ethical challenges experienced establishing and implementing a managed access data-sharing mechanism.

### Resource Implications

Staffing the data management team and obtaining data management training was especially difficult. This was primarily due to a lack of local expertise for data management because of the following: industry-sponsored trials in this region do not typically conduct their data management locally, the career path for data managers is unclear and there is a lack of availability of formal data management training.

In addition to human resources, a robust data management system and relevant hardware was required. Many study sponsors and funding agencies request that the data management system is compliant with the Good Clinical Practice standards, US FDA Title 21 Code of Federal Regulations Part 11 requirements which are as follows: system validation, robust audit trail, security access control, specification for system design and edit checks, archiving procedures and electronic signatures. These sophisticated systems are expensive and constitute a significant proportion of any clinical trial budget.

In order to enable sharing of datasets, additional resources were required of the data management team, data access committee members and legal and clinical trial teams. A significant amount of time was required for communications and administrative purposes. For example, for every dataset shared, a data manager had to answer several emails explaining the dataset and the relevant metadata.

These additional time and infrastructure requirements had significant resource implications. We have discussed establishing a cost recovery mechanism but have yet to come to a conclusion. Discussions on this topic include ethical considerations such as the fine line between cost recovery and commoditisation of health research data.

### Consent

For many datasets applied for, neither prior-specific consent nor broad consent to data sharing had been obtained as they were collected prior to the implementation of data-sharing policies and processes at MORU. For these datasets, the DAC approved the request on the grounds that there was minimal risk to the data subjects, the potential benefits of sharing the datasets outweighed the potential risks, and that it was impracticable to go back to participants to request consent to sharing. A seminal example is a severe malaria study comparing artesunate and quinine therapy involving more than 5000 African children conducted between 2005 and 2010 (Dondorp et al. 2010). Falciparum malaria is still a major contributor to child mortality in Africa and one of the main causes of paediatric hospital admission across sub-Saharan Africa. It is ethically unacceptable to repeat the study as the trial had provided strong evidence that artesunate was superior to quinine in the treatment of severe malaria. This dataset is a valuable resource for any researcher working on severe malaria treatments.

‘Broad consent’ has been proposed as a mechanism to enable potential research participants to give permission for their data to be used in future research studies (CIOMS 2016). Proponents of broad consent argue that broad consent can be considered ‘informed’ consent and is justified by appeal to the principle of respect for autonomy (Sheehan 2010). The argument is that broad consent is a decision to allow others to decide and a consent to a process of governance provided that the governance structure is robust and trustworthy (CIOMS 2016; Sheehan 2010).

From our experience conducting studies in low-income settings, participants rarely fully comprehend the information in the primary studies (Das et al. 2014). Data sharing is a concept that is removed from the daily lives of many of our participants. Providing information on data sharing and obtaining broad consent for data sharing in addition to the consent for the primary study adds a layer of complexity to the consent process.

While ethical arguments for and against broad consent have been extensively debated in the literature, there has been less consideration of how best to explain data sharing and its governance when obtaining consent for future use of data. A qualitative study is underway at MORU to better understand these challenges and how to best to obtain such consent.

### Applicants and Proposed Projects

Although the majority of MORU research data is generated in LMICs, to date, no requests for access to MORU data have been received from institutions in LMICs. Instead, applicants tend to be from well-resourced groups in higher-income settings who have good IT infrastructure and the capacity to conduct complicated statistical analyses and mathematical modelling. Concerns have been raised that data-sharing policies and processes should minimise exacerbation of current inequities between higher- and lower-income settings (Bull 2016; Bull et al. 2015a, b). A lack of applications for MORU data from LMICs illustrates the need for capacity building in data management and data analysis, so that researchers from such settings are not just able to share data, but also able to access datasets and conduct their own secondary analyses.

The DAC has received applications from three different pharmaceutical companies for data from trials conducted in LMICs for purpose of supporting the registration of products in developed countries. The MORU data-sharing policy does not prohibit sharing of data with commercial companies. During the review of these applications, the background of the company and the potential benefits to the communities from which the data were obtained were discussed. These applications were particularly challenging as the DAC is neither equipped nor resourced to conduct thorough background checks on companies, or to comprehensively assess the potential benefits of such uses of data. These companies also asked for accompanying documents required for new drug application, which were not available as the trials were not designed as regulatory trials.

### Data Access Committee

The current DAC is composed of senior members of MORU including researchers, the head of data management and the Chief Operating Officer. The Council for International Organizations of Medical Sciences (CIOMS) 2016 guidelines suggest that governance structures should have ‘representation of the original setting’. We agree that it could be good to include community representation but questions remain about the practicalities of doing so and who counts as a representative of the original setting. An alternative to representatives could be to take a similar approach to research ethics committees and include independent lay members on the DAC. The DAC is currently considering how best to have insight from research populations during its review process.

## Research Agenda

The recent CIOMS guidelines suggest a set of good practices for collection, storage and use of data in health-related research. They are useful as a starting point and address many important issues such as consent, governance structures and confidentiality. From our experience, trialling a data-sharing policy and reviewing access requests, we have identified a number of research questions and gaps in these recommendations. The research agenda below is proposed to answer some of these lacunae.

1. There is a need to identify the infrastructure and processes required to promote data sharing for all research and the costs of implementing these, including in LMICs. Priority areas include data management and IT needs such as data management personnel, software, repositories, data documentation standardisation and de-identification techniques. A comprehensive tool kit that includes templates for data-sharing policies, data management policies, DAC terms of reference, data access agreements, data management budget templates and an indication of the basic human and technical infrastructure needs to be developed. These can be adapted and adopted by research groups to suit their contexts.
2. A gap analysis should to be conducted to identify training needs to inform the development of specialised training materials for building capacity to curate and manage data for sharing and to conduct secondary analyses on available data. These training materials should be accessible to all researchers including those in LMICs.
3. Social science research is necessary to understand key stakeholders’ experiences in data sharing and key drivers and barriers to data sharing. Mixed methods research comprising qualitative interviews, focus group discussions and questionnaires should be conducted with researchers, data managers, administrators, data access committee members, ethics committee members and research participants, to address these issues.
4. Much has been written about the perceived benefits and risks of data sharing but there is a paucity of empirical data about benefits and risks in practice. Shared datasets should be tracked and in-depth case studies conducted to document to the impact of data sharing. These should include evaluation of outputs of data sharing including academic papers, documented amendments to disease management and health policies, and related benefits or harms.
5. DACs have been developed relatively recently. Many questions still remain unanswered such as appropriate methods to constitute a DAC, means of including representation of the research setting, and how to conduct a review of potential benefits and risks of sharing data. Studies should be conducted with DACs to track the type and objectives of applications received, geographical spread of applicants, concerns raised by the DAC and potential means of responding to these.
6. There are a number of research questions around consent for data sharing. Qualitative research should be conducted to answer questions such as perceptions and attitudes towards broad consent, how much information should be provided on the governance structure to participants and how best to explain data sharing comprehensibly.

## Conclusions

In this paper, we share our experience establishing the requisite mechanisms for data sharing, which include good data management systems and data-sharing infrastructure and incorporating a data-sharing policy, the DAC and related procedures. We experienced many practical and ethical challenges during the first 18 months of operating a data access procedure, including requests for datasets without prior specific or broad consent to sharing and the complexities of evaluating requests from pharmaceutical companies. We also encountered significant resource implications, which required the development of human resources and infrastructure. We conclude by suggesting a research agenda to promote responsible and equitable data sharing.
